# 1139. Reducing Collection of Tracheal Aspirate Bacterial Cultures: A Diagnostic Test Stewardship Intervention

**DOI:** 10.1093/ofid/ofab466.1332

**Published:** 2021-12-04

**Authors:** Kathleen Chiotos, Giyoung Lee, Guy Sydney, Heather Wolfe, Jennifer Blumenthal, Hannah Stinson, Nancy McGowan, Julie Harab, Danielle Traynor, Aaditya Dudhia, Joseph Piccione, Jennalyn Burke, Ashley Doll, Garrett Keim, Charlotte Woods-Hill, Megan Jennings, Rebecca Harris, Jeffrey Gerber

**Affiliations:** Children’s Hospital of Philadelphia, Philadelphia, Pennsylvania

## Abstract

**Background:**

Tracheal aspirate (TA) bacterial cultures are often collected in mechanically ventilated children to evaluate for ventilator-associated infections (VAI), including tracheitis and pneumonia. However, frequent bacterial colonization of tracheal tubes results in poor specificity of positive TA cultures for distinguishing bacterial infection from colonization, which contributes to antibiotic overuse for VAI. We performed a quality improvement project to reduce collection of TA cultures through implementation of a consensus guideline to standardize culture ordering, and measured its impact on antibiotic use in a tertiary PICU.

**Methods:**

A multidisciplinary team including PICU, pulmonary, and ID clinicians developed the consensus guideline in November 2019-February 2020. The first Plan-Do-Study-Act (PDSA) cycle occurred in August 2020 and included provider education, providing a link to the guideline in the TA culture order, and signs and screensavers highlighting key guideline recommendations. The second PDSA cycle occurred in October-December 2020 and included weekly emails to on service PICU clinicians. Statistical process control charts were used to measure the number of TA cultures collected/100 ventilator days and broad-spectrum antibiotic DOT/100 ventilator days. The number of patients treated for VAI/100 ventilator days and guideline compliance were also measured.

**Results:**

The baseline rate of TA culture collection was 4.58/100 ventilator days. A centerline shift to 3.33 cultures/100 ventilator days occurred in March 2020. Following PDSA 1 and 2 in October 2020, a second downward centerline shift to 2.22 cultures/100 ventilator days occurred (Figure 1). Broad-spectrum antibiotic days of therapy/100 ventilator days decreased in November 2019 coincident with the start of the project, but no further reductions occurred after PDSA 1 and 2 (Figure 2). The number of patients treated for VAI decreased from a baseline of 1.24/100 ventilator days to 0.66/100 ventilator days. Finally, the proportion of TA cultures ordered that were non-compliant with the guideline recommendations was unchanged throughout the study period (Table 1).

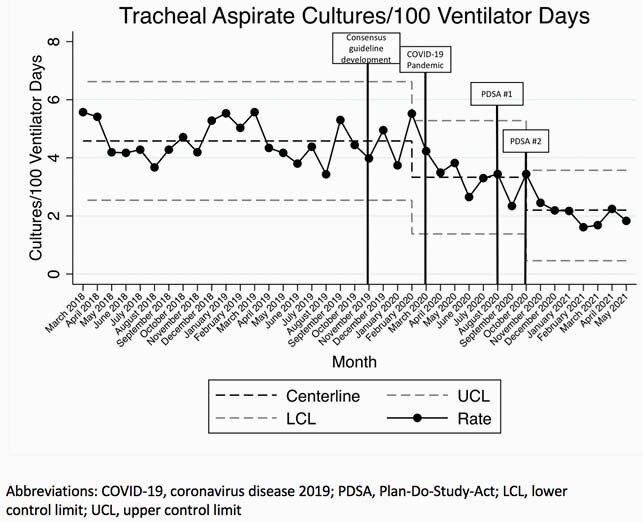

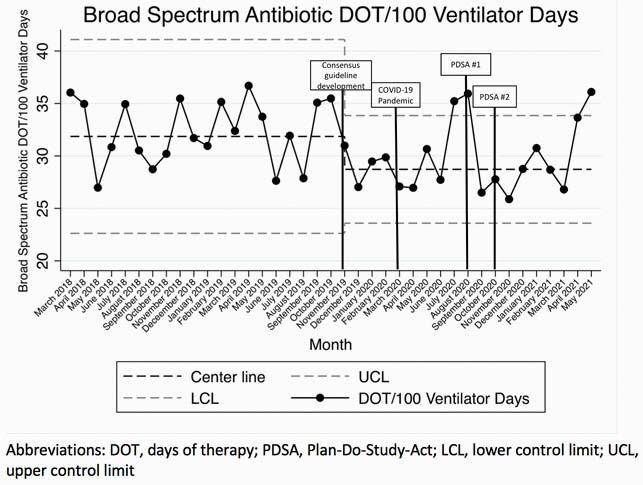

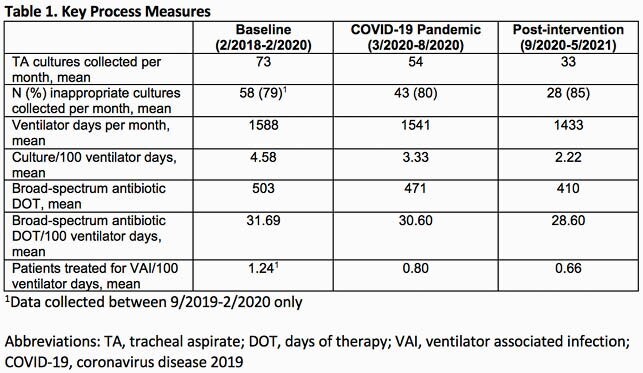

**Conclusion:**

A consensus guideline reduced collection of TA cultures, with a modest reduction in the rate of antibiotic treatment for VAI.

**Disclosures:**

**All Authors**: No reported disclosures

